# In silico comparative genomic analysis of GABA_A _receptor transcriptional regulation

**DOI:** 10.1186/1471-2164-8-203

**Published:** 2007-06-30

**Authors:** Christopher J Joyce

**Affiliations:** 1 Faculty of Biological Sciences, The University of Leeds, Leeds, UK

## Abstract

**Background:**

Subtypes of the GABA_A _receptor subunit exhibit diverse temporal and spatial expression patterns. *In silico *comparative analysis was used to predict transcriptional regulatory features in individual mammalian GABA_A _receptor subunit genes, and to identify potential transcriptional regulatory components involved in the coordinate regulation of the GABA_A _receptor gene clusters.

**Results:**

Previously unreported putative promoters were identified for the β2, γ1, γ3, ε, θ and π subunit genes. Putative core elements and proximal transcriptional factors were identified within these predicted promoters, and within the experimentally determined promoters of other subunit genes. Conserved intergenic regions of sequence in the mammalian GABA_A _receptor gene cluster comprising the α1, β2, γ2 and α6 subunits were identified as potential long range transcriptional regulatory components involved in the coordinate regulation of these genes. A region of predicted DNase I hypersensitive sites within the cluster may contain transcriptional regulatory features coordinating gene expression. A novel model is proposed for the coordinate control of the gene cluster and parallel expression of the α1 and β2 subunits, based upon the selective action of putative Scaffold/Matrix Attachment Regions (S/MARs).

**Conclusion:**

The putative regulatory features identified by genomic analysis of GABA_A _receptor genes were substantiated by cross-species comparative analysis and now require experimental verification. The proposed model for the coordinate regulation of genes in the cluster accounts for the head-to-head orientation and parallel expression of the α1 and β2 subunit genes, and for the disruption of transcription caused by insertion of a neomycin gene in the close vicinity of the α6 gene, which is proximal to a putative critical S/MAR.

## Background

**Figure 1:**
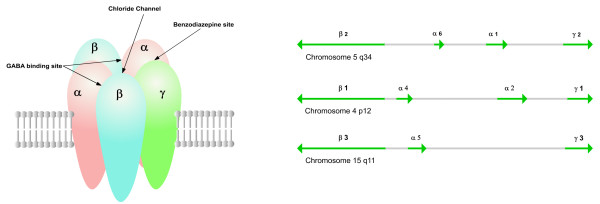
**(a) GABA_A _receptor subunit composition (b) GABA_A _receptor gene clusters**. (a) Two α, two β and one γ subunit surround a central channel, with a GABA binding site located at each α-β interface. (b) Gene order, intergenic distance and the head-to-head orientation of the α and β subunit genes are conserved in each cluster. After Russek [8]

The GABA type A (GABA_A_) synaptic receptor is a ligand-gated ion channel (LGIC), an integral membrane protein which mediates fast synaptic transmission. It is a member of a superfamily of synaptic receptors which also includes the nicotinic acetylcholine receptors (nAChR). All of these family members are membrane-spanning pentamers, the five homologous subunits surrounding a central channel (figure 1).

There exist at least 16 GABA_A _receptor subunit isoforms in mammals, each encoded by a separate gene. These isoforms have been categorised into classes based upon sequence similarity: six in the α subunit class, three β, three γ, and one each of δ, ε, θ and π. Typically two α, two β and one γ subunit assemble to form the GABA_A _receptor, with a GABA binding site located at each α-β interface. The most widely expressed and most common receptor subtype is a combination of two α type 1 subunits with two β type 2 and one γ type 2, which constitute in the region of 40% of receptors in the mammalian brain [1]. Alternatively spliced subunit variants also contribute to the diversity of GABA_A _receptor composition.

GABA_A _receptor subtypes are distributed differentially within both cell type and region in the CNS, some subtypes being widespread whilst others have a very restricted expression profile. This, and the observation that expression of receptor subtypes varies with developmental stages, indicates that they each fulfil specific physiological roles. Furthermore, the subunit composition of GABA_A _receptor populations is not static within regions of the adult CNS, and alterations of subunit expression are observed in response to exposure to a large number of neuroactive compounds [2].

These complexities in GABA_A _receptor subtype expression are determined primarily at the level of transcription, by cell-specific coordinate gene expression [3,4]. Typically, both tissue-specific and ubiquitous transcription factors are required to activate and control the expression of a gene. Many genes which are expressed only in the nervous system contain a 21-bp neuron-restrictive silencer element (NRSE) motif. This element binds with neuron-restrictive silencing factor (NRSF – also known as RE1 silencing transcription factor, REST) to repress gene expression. Since this factor is expressed primarily in non-neuronal tissues, the NRSE element acts as a gene silencer in these tissues. The GABA_A _subunit γ2 gene contains an NRSE site in the first intron, which was shown to bind to NRSF and repress expression in non-neuronal cell lines. There exist also NRSE-like sequences in the genes of GABA_A _subunits α1,α5,α6,δ and β3, each downstream of the TSS [5].

Generic cis-acting regulatory elements can also provide tissue specific transcriptional regulation by binding to factors that are present in a tissue-specific manner. The SP1 binding site is recognized by a family of transcription factors, and has similar binding affinity with SP1, SP3, and SP4 factors. Whilst SP1 and SP3 are ubiquitously expressed, SP4 is relatively brain-specific. SP3 acts as either an activator or a repressor, depending on promoter context. One possible explanation of the neural specificity of the α4 subunit promoter is that, in non-neuronal cells, factors SP1 and SP3 binds with the SP1 site in the proximal promoter to suppress transcription, whereas in neurons SP3 and SP4 bind at the site to activate transcription [6].

Alternate splicing of the GABA_A _receptor subunit gene transcripts provides for mRNA subunit variants. The α2 subunit, for example, exhibits a complex pattern of alternative splicing, with distinct promoter regions for the alternative mRNA isoforms. Although the resulting encoded protein sequence is the same, variations in the stability of mRNA isoforms can affect translational efficiency; these variations and the use of alternative promoters allow fine control of α2 subunit expression subunit during brain development [7]. Multiple promoters exist also for the α5 and β3 subunit genes, and provide for further diversity in their transcriptional and post-transcriptional control.

### GABA_A _receptor subunit gene clusters

The genes encoding for vertebrate GABA_A _receptor subunit genes are organised into several gene clusters on different chromosomes, which appear to have arisen due to duplication and subsequent translocation of an ancestral gene cluster. Two clusters on human chromosomes 4 and 5 are composed of two α genes, one β, and one γ (Figure 1). Another cluster on chromosome 15 is composed of one α, one β and one γ gene [8]. Evidence that the clusters have arisen by means of gene duplication events is provided by the conservation of gene order, intergenic distance and the head-to-head orientation of the α and β subunit genes in each cluster. Based on conservation of genomic organization of GABA_A _receptor gene clusters, the ε and γ subunit genes appear to have a common ancestor.

This organisation into clusters may have been preserved to provide a mechanism for facilitating coordinate gene expression, by allowing adjacent clustered genes to share regulatory elements. Genes in clusters may be co-regulated in part by the establishment of chromatin domains which are insulated from surrounding genetic material by chromatin boundaries. The genes for the components of most common GABA_A _receptor subunit configuration, α1-β2-γ2, lie within the same cluster on chromosome 5, along with the α6 gene. All of the genes in this cluster are highly expressed, at least in granule cells, in contrast to the genes clustered on chromosomes 4 and 15, suggesting a common transcriptional regulatory mechanism. However, the α6 gene has a much more restricted pattern of expression, showing that it clearly also retains independent regulatory features.

### Coordinate gene regulation and locus control regions

Co-regulated genes may each possess a copy of the same transcriptional regulatory features, or they may share common distal features such as enhancers [9] or Locus Control Regions (LCRs)[10]. LCRs are regions of genomic DNA which are able to exert long-range enhanced transcription of linked genes in a tissue-specific and copy-number dependant manner (i.e. proportionate to the number of gene copies in transgenic experiments). They are composite features, characterised biochemically by a series of DNAse I hypersensitive sites (HSs), some of which contain arrays of transcriptional factor binding sites [10]. There is no single model for the long-range transcriptional control exerted by LCRs. They are thought to both possess classic enhancer activity, recruiting the transcriptional machinery to gene promoters, and to play a role in establishing and propagating chromatin opening. LCRs are composed of varying numbers of both tissue-specific HSs and non-cell specific HSs, some of which are non-functional, others fulfilling distinct roles in the augmentation of transcription. The HSs that constitute an LCR are not necessarily clustered in a region of 1–2 kb, as they are in the canonical human β- globin gene locus [11]. They may consist of HSs spread over large distances, and be interspersed with the genes they control.

Experimental techniques (RNA TRAP and 3C) have verified that the human β-globin LCR directly comes into direct contact with the DNA of the expressed gene whilst the locus is transcriptionally active, with intervening DNA looping out and away from the active region [12]. This contact presumably facilitates chromatin-remodelling activity in the vicinity of the gene, and also allows the LCR enhancer elements and proximal promoter to cooperatively recruit transcriptional machinery proteins, as per the classic model for enhancer function [13]. There is evidence that the human β-globin LCR interacts with only one gene promoter at a time and that it may alternate between two or more promoters, depending on the stage of development [10].

LCRs, unlike simpler enhancer elements, operate in an orientation-dependent manner with respect to the genes they control [11]. It seems improbable, therefore, that a canonical LCR could control both the α1 and β2 genes, which unlike the genes in other LCR-controlled clusters, are oriented in a head-to-head configuration. It seems more likely that these genes are co-regulated by shared elements which are not orientation specific in relation the genes they activate. Some genes migrate beyond their immediate chromosomal territories to transcription factories, which are shared sites of transcription enriched in RNA Pol II and transcriptional factors. Transcriptional enhancer elements located in between the α1 and β2 genes could exert their effects on both genes, in a transcription factory located outside the of immediate chromosome territory ([13]. The chromatin of the coordinately expressed genes in the HoxB cluster locates in loops which extend outside of the chromosomal territory in expressing cells, but not in non-expressing cells [14].

**Figure 21:**
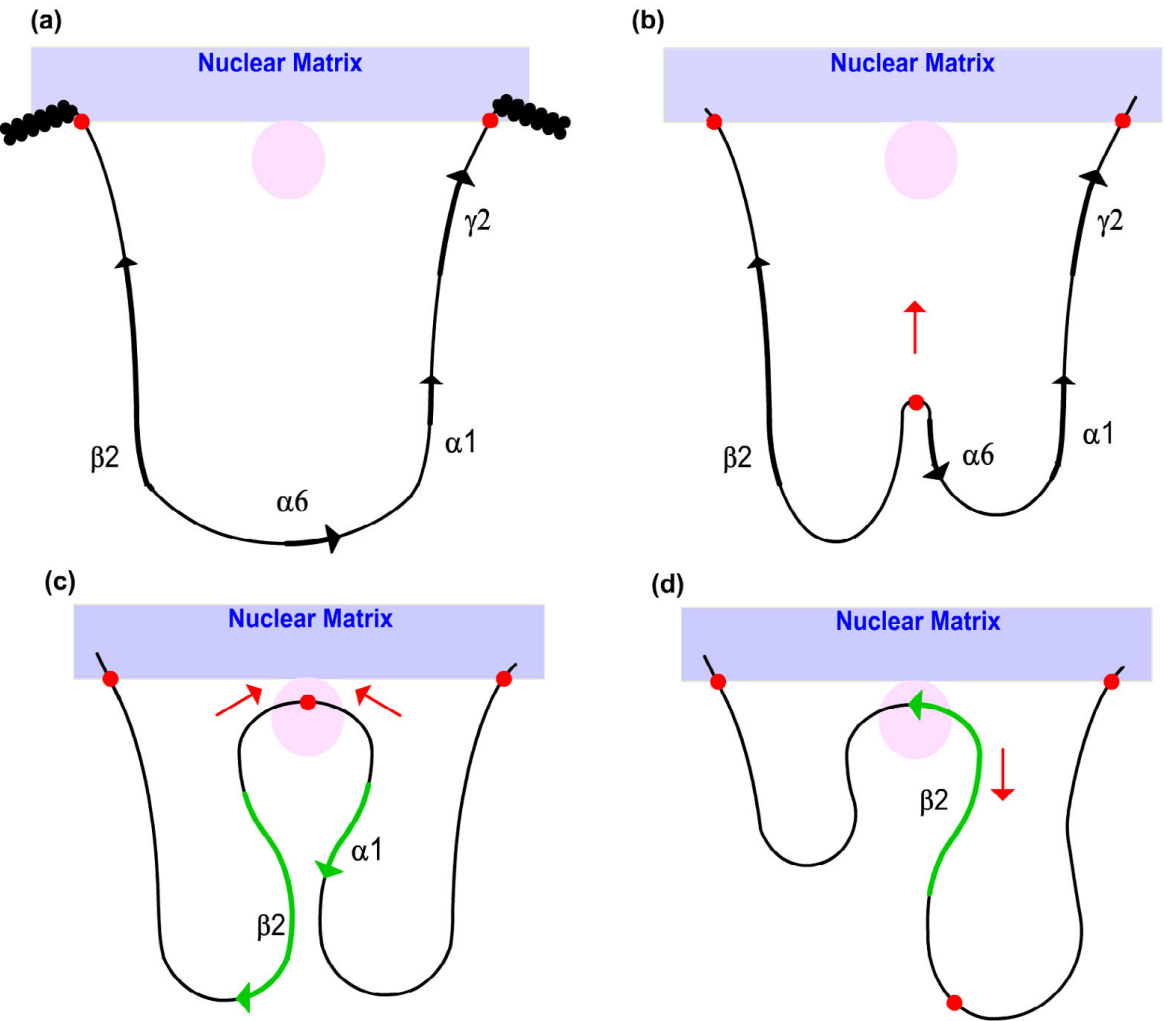
**Model for S/MAR associations in GABA_A _receptor subunit gene locus**. (a) Gene locus anchored to nuclear matrix by flanking S/MARS (red) establishes open chromatin conformation. (b) Functional S/MAR mediates loop attachment to transcriptional machinery (pink). (c) Chromatin is reeled through transcriptional machinery in either direction to activate transcription of β2 or α1 gene (d) functional S/MAR dissociates upon elongation. (After Martins et el [15], Heng et al [17]).

One model which reconciles the unrestricted activity of enhancers implied by in the familiar looping model with the positional effects observed in silencer elements, suggests that the enhancer first directs the assembly of, or migration to, a transcription factory, and then reels in the chromatin in search of a promoter. This model explains how silencer elements could prevent gene access to the transcriptional factory only if they are positioned between enhancer and promoter [13] (see figure 21).

### Chromatin dynamics and scaffold/matrix attachment regions

Chromatin is a dynamic structure that modulates access to DNA during gene replication and transcription. Whereas housekeeping genes are generally partitioned into chromosomal segments which are constitutively in an open conformation, tissue-specific genes tend to exist in segments which are facultatively opened [15]. Groups of genes may be regulated autonomously by organising into chromatin domains, which are maintained independently from their surroundings and demarked by dynamic chromatin boundaries. Several models have been proposed to explain how chromatin boundaries are established. Barrier activity may be accomplished by the creation of a nucleosome gap to interrupt repressive histone modification, or barrier proteins may disrupt histone modifications associated with activation to prevent the propagation of heterochromatin [13]. It seems clear that chromatin domains are maintained by the tethering of a loop of chromatin to a fixed structure, isolating it from propagating histone modification processes associated with chromatin condensation and transcriptional repression [16,17]. In eukaryotes, this structure is the nuclear matrix (or nuclear scaffold), a network of proteins that provides a framework for organising chromatin. Filaments of the nuclear matrix provide structural support for the formation of loops of DNA during replication and transcription. Attachment of genomic DNA to the nuclear matrix also places genes in the domain in proximity to chromatin-modifying complexes and the transcriptional factors which enhance expression. Scaffold/Matrix Attachment Regions (S/MARs) are eukaryotic DNA sequences capable of specific binding to nuclear proteins that are part of the nuclear matrix. Their interactions with the nuclear matrix provide anchor points from which chromatin loop domains can be established and maintains the open chromatin conformation necessary for transcription.

The use of S/MARs as nuclear matrix anchors occurs in vivo in a discriminative and tissue-specific manner [17]. Experimental evidence suggests that a subset of S/MAR sequences may function as relatively static nuclear matrix anchors, whilst other S/MARs act more dynamically to draw the chromatin loop into transcriptional machinery on the nuclear matrix surface, which is then scanned in search of promoters using a chromatin reeling model, thus promoting transcriptional initiation (figure 21). A distinction is made between the relatively fixed *structural *S/MARs serving as anchors, and *functional *S/MARS which operate dynamically under the control of the transcriptional regulatory system to draw genes into the transcriptional machinery ([17]. S/MARS also may play a role in demethylation of DNA regions to trigger chromatin opening. It seems reasonable to hypothesise that the coordinate and tissue-specific regulation of genes in the GABA_A _receptor clusters may be achieved at least in part by the S/MAR-directed establishment of chromatin domains.

### Down-regulation of α1 and β2 expression in α6 knockout mice

In two separate experiments, the insertion of neomycin hybrid genes into exon 8 of the α6 subunit gene region brought about a parallel reduction in the expression of α1 and β2 in the forebrain of α6 subunit gene knockout mice [18]. Expression of γ *2 *mRNA was unaffected by the neomycin gene insert. This suggests that the insert may have interfered with one or more long-distance regulatory features directing the coordinate expression of the α1 and β2 subunit genes. This feature could be an enhancer or an LCR common to both genes and responsible for their observed parallel expression profiles. Long-range disruption of neighbouring genes was also demonstrated by the insertion of the PGK-Neo hybrid gene into the granzyme B and β-globin gene locus, which also reduced expression of multiple genes within the locus at distances greater than 100 kb [19].

Any model proposed for the coordinate regulation of α1 and β2 subunit gene expression needs to incorporate the observation that their expression is down-regulated upon insertion of the neomycin gene in the vicinity of the α6 subunit gene. Uusi-Oukari et al [18] hypothesise that the inserted sequence may contain cis-acting elements which interact with an LCR to reduce its effectiveness as an enhancer of α1 and β2 transcription. The presence of an addition 2 kb or 5 kb of sequence could also critically alter chromatin loop configuration or chromatin remodelling processes; or the neomycin gene inserts in the vicinity of an enhancer could disrupt assembly of, or migration to, a transcription factory, causing a parallel decrease in expression of both genes.

### Bioinformatics analysis of GABA_A _receptor subunit transcription regulation

Investigation of the transcriptional regulation of GABA_A _receptor subunit genes can provide an understanding of mechanisms underlying the spatial and temporal expression patterns of GABA_A _receptor subunit expression, which contribute to the aetiology of a wide range of neurological disorders and their responses to drug treatment. Steiger & Russek [20] have conducted a comprehensive review of GABA_A _receptor subunit gene regulatory features. In addition to summarising experimentally determined promoters, they used the Matinspector and NNNP programs to predict additional promoter regions and transcription factor binding sites. However, the published predictions were based upon analysis of a single (mostly human) genome for each subunit.

The purpose of the current work was to utilise a wide range of promoter prediction software to analyse each mammalian GABA_A _receptor subunit gene, and to use comparative genomic analysis to further substantiate the predictions made by these tools. Comparative analysis can serve to eliminate false positives from the large result sets typically created by Transcription Factor Binding Site (TFBS) prediction programs, and to provide powerful evidence for the functionality of putative TFBSs. Functional TFBSs sites are likely to be the conserved, and to be located in equivalent positions in multiple sequence alignments of homologous sequences, whereas false positives are not. Additionally, this work attempts to look beyond the regulatory elements for individual genes to propose models for higher level of transcriptional regulation of clustered GABA_A _receptor genes, focusing upon the coordinate expression of genes in the α1, β2, γ2 and α6 subunit cluster. The methods used for predicting features responsible for coordinate expression included comparative analysis of the cluster to reveal homologous regulatory features in intergenic regions of DNA, prediction of distal regulatory features as regions of DNase hypersensitivity, and prediction of Scaffold/Matrix Attachment Regions (S/MARS).

## Results and discussion

### GABA_A _α1 subunit gene

The α1 subunit gene is strongly expressed in all brain regions, being a component of the most abundant GABA_A _receptor subtype, α1β2γ2 [21]. Kang et al [22] have experimentally isolated a 60 bp minimal promoter element in the 5' flanking DNA sequence of the human α1 subunit gene. It was demonstrated that reduction in transcription of the subunit gene following chronic benzodiazepine exposure is brought about by direct repression of the activity of this promoter. Bateson et al [23] have identified the TSS for the chicken GABA_A_α1 subunit gene, and reported a number of putative promoter elements, including a TATA box 30 bp upstream of the TSS, SP1 binding site and reverse CCAAT box. The promoter region is CG-rich. The TSS for the rat gene has also been experimentally determined, and lies 5 bp 3' of the chicken TSS (A.N. Bateson, personal communication, unpublished).

**Figure 2:**
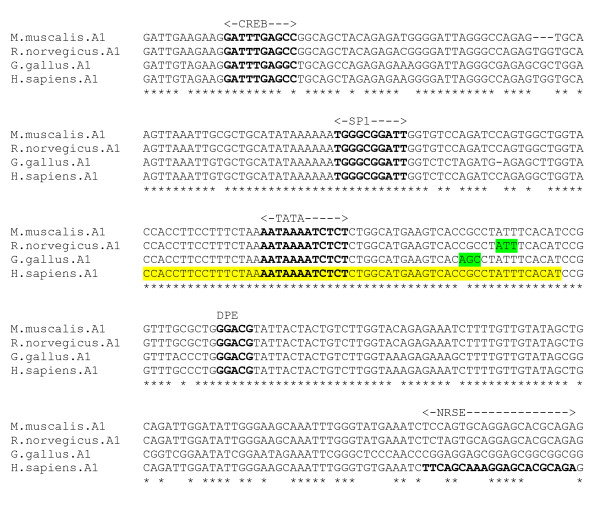
**α1 gene alignment, core promoter region**. Experimentally determined chicken and rat TSS (green), and human promoter region (yellow) highlighted.

An alignment was produced from the 5' flanking genomic DNA sequence of the α1 receptor for four species – human, chicken, mouse and rat (Figure 2). This alignment reveals a highly conserved section, across all species, corresponding to the experimentally determined human promoter region, and the chicken and rat TSS. Sequence identity is almost 100% in the core promoter region. Sequence data for more evolutionarily distant species, with sufficient identity to reveal any crucially conserved motifs within more divergent regions of sequence, was unavailable for the α1 subunit gene. The chicken TSS *AGC *is unique in the alignment, replaced with *CGC *in the mammalian species. There exists cDNA sequence for the mouse α1 gene which extends at 137 nt upstream relative to the chicken TSS [23]. This suggests the presence of a TSS upstream of that identified for the chicken, and the possibility of Multiple Start Sites for this gene. There is unpublished evidence that this is the case [24]. However, a scan for promoter regions using the PromoterInspector program did not identify any putative promoter region upstream of the mouse cDNA sequence. The TATA box and SP1 site [23] are fully conserved. In addition, a DPE element *GGACG *is conserved in its precise position with respect to the chicken TSS (28 bp downstream). There is also a conserved cAMP Response Element (CREB) element upstream of the minimal promoter, detected by the P-MATCH program. An NFI motif *CTCTGGCATGAAGTCACA *lies between TATA box (partially overlapped) and TSS. An NRSE-like sequence *TTCAGCAAAGGAGCACGCAGA *in the human gene lying approximately 115 bp downstream of the TSS [20] is not conserved across species.

### GABA_A _α2 subunit gene

The α2 subunit gene is strongly expressed only in the hippocampus and hypothalamus. Enhanced mRNA levels occur early in CNS development, and decline postnatally with a concurrent rise in α1 gene expression [7]. Fuchs & Celepirovic [7] have investigated the rat α2 subunit gene, and identified six α2 subunit mRNA isoforms with distinct 5-end UTRs (the resulting protein sequence is not affected), generated from three alternative first exons by means of alternative splicing and alternate promoter usage. The isoforms are named AB8, SPL2, SPL3, SPL4, SPL5 and non-spliced variant nonSPL. About 70% of expressed mRNA is of the AB8 isoform. Multiple Starts Sites (MSS) for each isoform were identified within each of the three alternative first exons. The AB8 isoform putative promoter region is GC-rich, and contains putative INR elements and TF binding sites, including SP1 binding sites. Unusually, the AB8 promoter region is located downstream from the transcription initiation sites [7].

**Figure 3:**
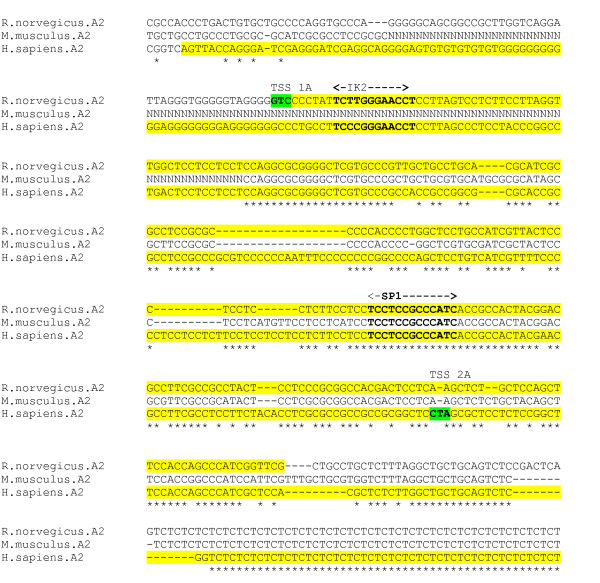
**α2 gene promoter alignment**. Experimentally determined rat and human promoter regions in human exon 2A and rat exon 1A (yellow), with TSS (green). ('N' in sequence indicates undetermined nucleotide).

The human α2 subunit gene also exhibits a complex pattern of alternative splicing and alternative promoter use [24]. There are four major isoforms composed using combinations of alternative exons. Two alternative 5' exons each lie downstream of two different promoter regions. The alternative first exons and their promoters all lie within a CpG island as detected by CPGPlot. MSS genes are characterised by a transcription initiation window of weak transcription start sites, and their promoters characteristically lack a TATA box. These characteristics are apparent from an analysis of the putative core elements in the identified rat and human promoter regions; the sequences are largely devoid of TATA boxes or other canonical core promoter motifs. An alignment was produced for the region encompassing the human exon 2a TSS and promoter region, and the homologous rat Exon 1A promoter region, for 3 species – human, mouse and rat (figure 3). The experimentally determined TSS sites for human and rat do not coincide. There are fully conserved IK2 (Ikaros) and SP1 sites within the region.

### GABA_A _α3 subunit gene

The α3 subunit gene is expressed throughout the adult brain. Like the α2 gene, enhanced expression occurs early in CNS development and declines postnatally with an accompanying rise in α1 expression [25]. The promoter region and major TSS of the mouse α3 subunit gene have been experimentally determined [25]. The highly unusual promoter region contains a series of *GA *repeats about 40 bp upstream from the major TSS, which bind unspecified nuclear proteins and directly augment transcription, and in which lie several minor TSS sites. An adjacent series of three *GC *repeats, forming part of a putative E2F sequence motif, appear to also contribute to promoter activity.

**Figure 4:**
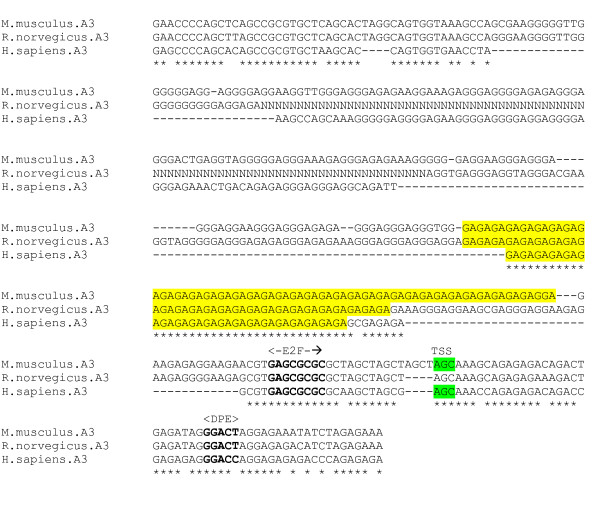
**α3 gene promoter alignment**. Experimentally determined mouse GA-repeat promoter region and homologues (yellow), with TSS in green. Putative TFBS sites in bold. (CLUSTAL-X gap extend penalty = 1).

An alignment was produced from the 5' flanking genomic DNA sequence of the α3 subunit gene for 3 species – human, mouse and rat (figure 4). The *GA *repeat regions, corresponding to the experimentally determined mouse promoter region, are of variable length across species [25]. The major TSS is conserved in the alignment. The core promoter is apparently devoid of TATA boxes or INR-like sequences. There is, however, a conserved DPE motif *GGAC [CT] *located 29 bp downstream of the determined TSS. Searches for TFBSs in the larger region encompassing the *GA *repeat core promoter, using the TESS, P-MATCH and MatInspector programs did not reveal any conserved ubiquitous or neuronal TFBS motifs in the proximal region upstream of the TSS.

### GABA_A _α4 subunit gene

The α4 subunit gene is expressed variably in different regions of the brain. Elevated expression levels are observed in animal models of temporal lobe epilepsy, and during withdrawal from alcohol and progesterone treatment, suggesting that the subunit may play a role in neuronal hyperexcitability [6]. Ma et el [6] have determined the minimal promoter, major TSS and start codon *ATG *for the mouse α4 subunit gene. They also identified two SP1 sites within the proximal promoter region which are critical for high-level promoter activity in vivo, and bind to the TFs SP3 and SP4 to augment α4 subunit gene expression in neuronal cells. Other putative SP1, AP1, c-Myb, and E-box binding sites did not alter transcription levels when deleted. Roberts et al [26] have demonstrated increases in mRNA levels of α4, and in early growth response factor 3 (EGR3) expression, in response to induced epilepsy, accompanied by increased binding of EGR3 to α4 subunits in dentate granule cells. Also, EGR3 knockout mice exhibit reduced α4 hippocampal mRNA expression. This data demonstrates the role of EGR3 as a major regulator of α4 subunits, and implicates the factor in epileptogenesis.

**Figure 5:**
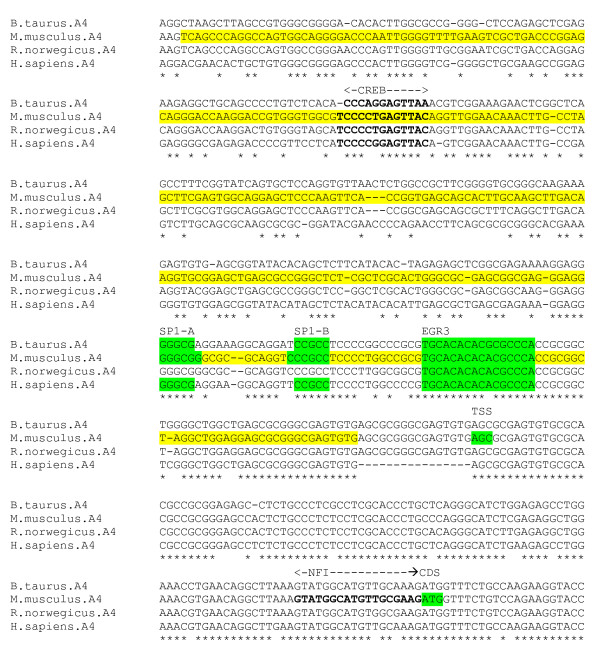
**α4 gene alignment, core promoter region**. Experimentally determined mouse CDS, TSS, EGR3 and SP1 sites (green), and minimal promoter region (yellow).

The promoter appears to contain no canonical core promoter elements. A predicted CpG island (CPGPlot) envelops the TSS. Recruitment of the basal transcription factors to form the pre-initiation complex presumably involves the SP1 sites in combination with one or more unknown promoter elements. An alignment of the promoter region for four species – human, cow, mouse and rat – reveals that the TSS, and SP1 and EGR3 sites are conserved across all species (figure 5). A motif *AGCGCGGGCGAGTGTG *occurs only once immediately prior to the TSS in the human sequence, but is exactly repeated in other species. A conserved CREB element lies within the minimal promoter, upstream of the SP1 sites. There is also a conserved NFI motif; however this lies outside of the identified mouse minimal promoter region, immediately upstream of the CDS.

### GABA_A _α5 subunit gene

Expression of α5 subunit receptors is restricted primarily to the hippocampus [21]. Kim et al [27] have identified three isoforms of human α5 subunit mRNA, resulting from three alternative first exons. They have demonstrated that each exon appears to be regulated by a different promoter, located in intronic sequence regions immediately upstream. The differential transcriptional activation by alternative promoters may determine the alternative usage of the first exon isoform. No core promoter element motifs are observed in these promoter regions, however both regions are enveloped within a single CpG island and contain a large number of putative TF binding sites including SP-1, AP-1 and AP-2 sites.

**Figure 6:**
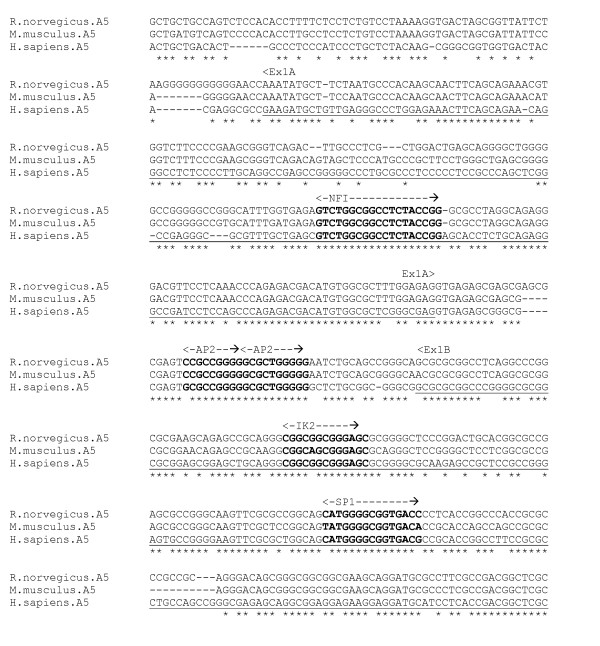
**α5 gene alignment, exons 1A and 1B core promoter region**. Experimentally determined human exons underlined.

An alignment was produced for human, rat and mouse sequences encompassing exons 1A, 1B, 1C and their associated promoter regions (figure 6). For the exon 1A promoter region, none of the putative binding sites identified [27] are conserved in the alignment; however there is a fully conserved NFI element within exon 1A, 124 bp downstream of the TSS. Within the Exon 1B promoter region lies a conserved sequence containing two overlapping reverse AP-2 motifs, and the exon itself contains IK2 and SP1 elements. Exon 1C and its promoter region are not well conserved across species, and none of the identified putative binding sites [27] are conserved in the alignment (data not show).

### GABA_A _α6 subunit gene

The α6 subunit gene is expressed exclusively in cerebellar granule cells [28], and as such provides a useful model for studying how neuron-specific gene expression is regulated. McLean et al [28] have identified several minor and one major TSS with a surrounding INR in homologous rat and mouse sequences. They have determined a 155 bp minimal promoter in the rat DNA, within which a 60 bp region, 70 bp upstream of the TSS, enhances expression in cerebellar granule cells only. This region is positionally dependent with respect to the TSS, and contains a conserved NF-1 (NFI, nuclear factor 1) motif. They have also identified a downstream negative regulatory region which is active in fibroblasts but inactive in cerebellar granule cells. Wang et al [4] have determined that NFI-A factor is abundant in cerebellar granule cells and binds to the α6 subunit gene promoter in vivo in the mouse gene, confirming its critical role in α6 subunit gene expression and the differentiation of cerebellar granule neurons. They have also identified the NFI motif *TGCCAAAAC *within the α6 gene promoter region which binds NFI-A proteins.

**Figure 7:**
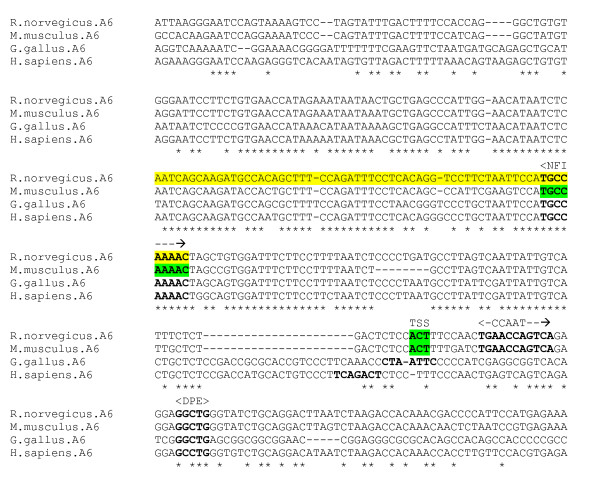
**α6 gene promoter alignment**. Experimentally determined rat minimal promoter (yellow), with rat and mouse TSS and mouse NFI site in green. Putative sites in bold.

An alignment of the promoter region for four species – human, chicken, mouse and rat – reveals that the rat and mouse INR motif encompassing the TSS [28] is not conserved in the human and chicken (figure 7), however the human sequence contains an INR-like motif *TCAGACT *9 bp upstream. The chicken sequence also contains a putative INR *CTAATTC *in the same region. A DPE element *GGCTG *at position +27 relative to mouse and rat TSS is conserved across species. For rat and mouse sequences, there exists a putative CCAAT box at position +10 relative to the TSS (not conserved in human and chicken). The NFI binding site [4] is fully conserved.

### GABA_A _β1 subunit gene

The β1 subunit is present in a high proportion of the GABA_A _receptors from the hippocampus and cerebral cortex, and a very small proportion of cerebellar GABA_A _receptors [29]. The human β1 subunit gene contains a TATA-less core promoter region of 270 bp [30]. The core promoter is neural specific and contains an INR element at the major TSS which is critical to promoter activity. Upstream of the INR, A GRE consensus sequence has a neural-specific positive regulatory effect, and CCAAT (NF-Y) and IK2 motifs have negative regulatory effects. Persistent receptor activation by the GABA neurotransmitter induces down-regulation of β1 subunit mRNA expression. The exposure to GABA appears to directly reduce promoter activity by modulating the binding of sequence-specific basal TFs to the promoters INR element, thus regulating formation of the Pre-Initiation Complex (PIC) [30].

**Figure 8:**
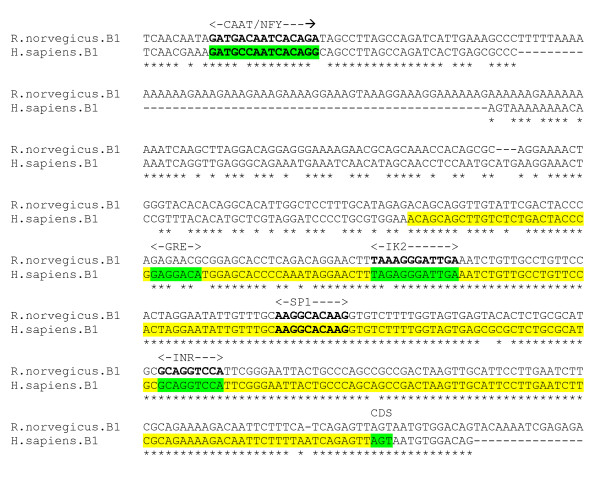
**β1 gene alignment, core promoter region**. Experimentally determined human minimum promoter (yellow), also INR, and GRE (positive) and CAAT and IK2 (negative) regulatory elements. Putative SP1 motif in bold.

Each of the experimentally determined regulatory elements are conserved in an alignment of human and mouse sequences encompassing the core promoter region (figure 8). The negative regulatory sequence reported as an IK2 binding site could also be interpreted as an OCT1 (octamer binding protein) motif *GGGATTGAAATCTG*. A conserved SP1 motif is situated within the identified minimal promoter, 50 bp upstream of the major TSS.

### GABA_A _β2 subunit gene

**Figure 9:**
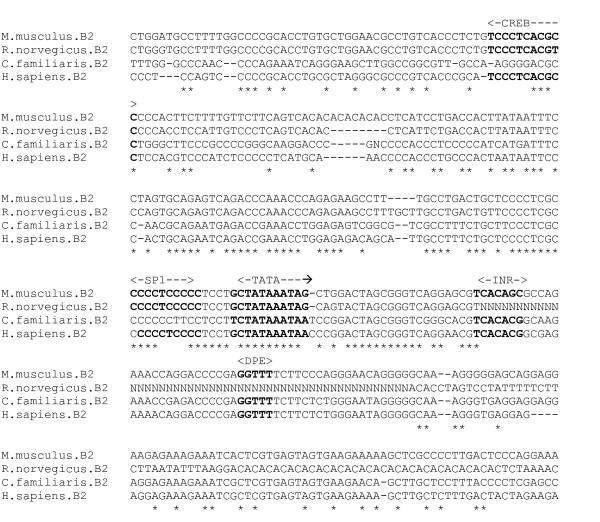
**β2 gene putative promoter alignment**. Putative core promoter elements in bold

The β2 subunit is a component of the most abundant GABA_A _receptor subtype, α1β2γ2, and is strongly expressed in all brain regions, having an almost identical expression profile to the α1 receptor. Alternative splicing of the gene generates two transcript variants, differing by a 114 bp insertion [31]. There is currently no experimental data available for transcription regulatory features in the β2 subunit gene. In order to search for a putative promoter region, GenBank DNA sequence for the human β2 subunit gene was searched using the programs PromoterInspector, NNPP, Promoter 2 and McPromoter, within a region extending from 2000 bp upstream of the mRNA start point to approximately 500 bp downstream. The results were compared and combined to derive a consensus predicted promoter region of 900 bp, 510 bp upstream of the GenBank CDS for further analysis. Homologous DNA sequences were then obtained for the β2 subunit gene in the mouse, rat and domestic dog species. This data was used to generate a multiple sequence alignment (figure 9), revealing a highly conserved region (78% for putative core promoter), containing putative TATA box, INR and DPE elements. Searches for TFBSs in the larger region encompassing the putative core promoter, using the TESS, P-MATCH and MatInspector programs did not reveal any fully conserved ubiquitous or neuronal TFBS motifs, however there is a partially conserved SP1 sequence and a CREB element upstream of the putative core promoter.

### GABA_A _β3 subunit gene

The β3 subunit is the most abundant β isoform in GABA_A _receptors of the hippocampus, and is also widely expressed in the cerebral cortex and cerebellum. It is frequently co-localised with either a β1 or β2 subunit in the same GABA_A _receptor, in proportions which vary with brain region [29]. The human β3 subunit gene contains two alternate first exons, 1 and 1A, which are expressed differentially during development and between different brain regions, and encode two dissimilar signal peptide-like sequences. Exon 1A lies upstream of exon 1 and encodes a peptide signal sequence. Transcription of exon 1A is initiated by multiple start sites in a pyrimidine-rich promoter region, which lies in between the alternative first exons, and binds SP1 and other nuclear factors. Nuclear factor binding sites overlap each of these transcription start sites, which may be involved in differential expression of the alternative first exons. The human and rat coding and promoter regions are highly conserved [32].

**Figure 10:**
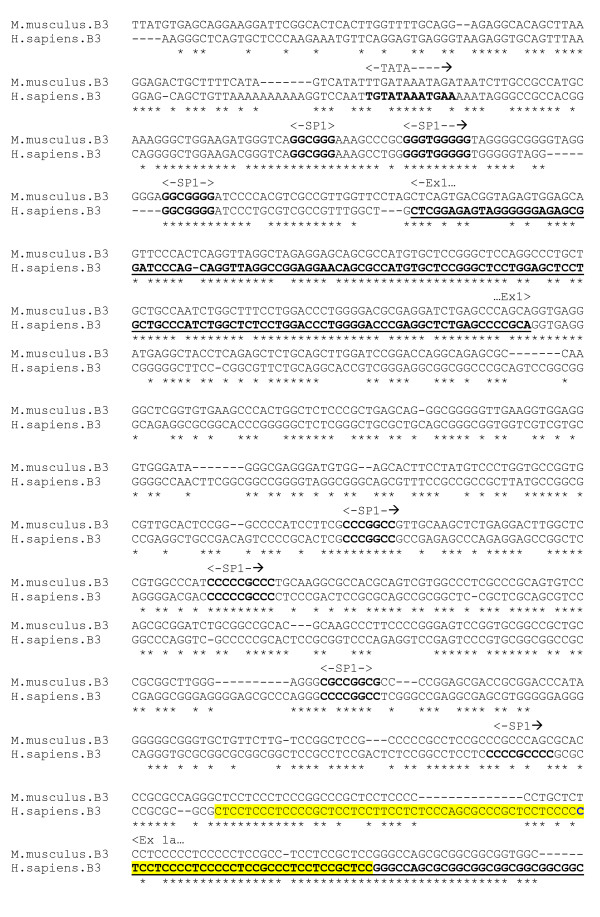
**β3 gene alignment, core promoter region**. Human alternate first exons underlined, pyramidine rich promoter (yellow). Putative SP1 elements in bold.

An alignment was produced of the human and mouse 5-end gene sequences, encompassing the upstream regions of the alternate first exons (figure 10). There are three conserved SP1 sequences upstream of exon 1, and a partially conserved TATA box. The exon 1A promoter region contains no canonical core promoter elements. This is not surprising given that it is composed almost entirely of pyramidines. Several conserved SP1-like sequences are located upstream from the pyrimidine-rich promoter region.

### GABA_A _γ1 subunit gene

**Figure 11:**
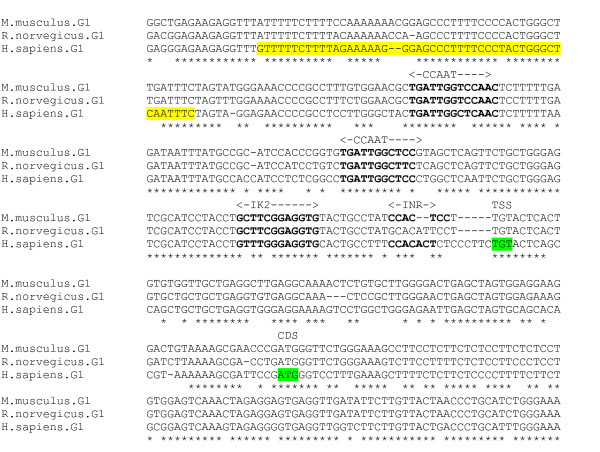
**γ1 gene putative promoter alignment**. Predicted human promoter (yellow) (NNPP) and GenBank TSS and CDS highlighted. Putative promoter elements in bold.

Expression of the γ1 subunit is more restricted than that of the γ2 subunit, occurring at high levels predominantly in certain amygdaloid, hypothalamic and premammillary nuclei [33]. The γ1 subunit gene promoter region has not been characterised. Analysis of the 2000 bp region upstream of the GenBank computationally-derived TSS for the human sequence by PromoterInspector and McPromoter predicted no promoter in this region; however the NNPP and Promoter 2 programs predicted promoter regions, 251–301 bp and 489–589 bp upstream of the GenBank TSS respectively. Homologous rat and mouse sequences for the human region encompassing the predicted promoter regions and GenBank TSS were obtained and aligned (figure 11). The GenBank putative TSS and CDS for each species is conserved in the alignment. An INR consensus sequence *CCACACT *is located 16 bp upstream of the human GenBank TSS. The region also contains an IK2 binding sequence and two conserved reverse CCAAT boxes, which could also be interpreted as NF1 binding sites.

### GABA_A _γ2 subunit gene

The γ2 subunit gene is expressed widely throughout the brain, occurring at high levels early on in CNS development [21]. Mu and Burt [5] have found two major transcription start sites in a TATA-less promoter region of the mouse γ2 subunit gene. There are two subtypes of the subunit, which are formed by alternate splicing which is regionally and developmentally regulated. The first intron of both the mouse and human γ2 genes contains a conserved neuron-restrictive silencer element (NRSE) site, which was shown to bind to NRSF and direct expression in neuronal cells, and to repress expression in non-neuronal cells. Another sequence element, the "Gamma Promoter Element" (GPE1), which also promotes expression in neuron-like cells, lies 70 bp downstream of the second major TSS. Mu and Burt [5] report that "probably only a portion of the 24 bp sequence is involved". The first 12 bp matches the cAMP-responsive element binding protein (CREB) consensus sequence (program P-MATCH).

**Figure 12:**
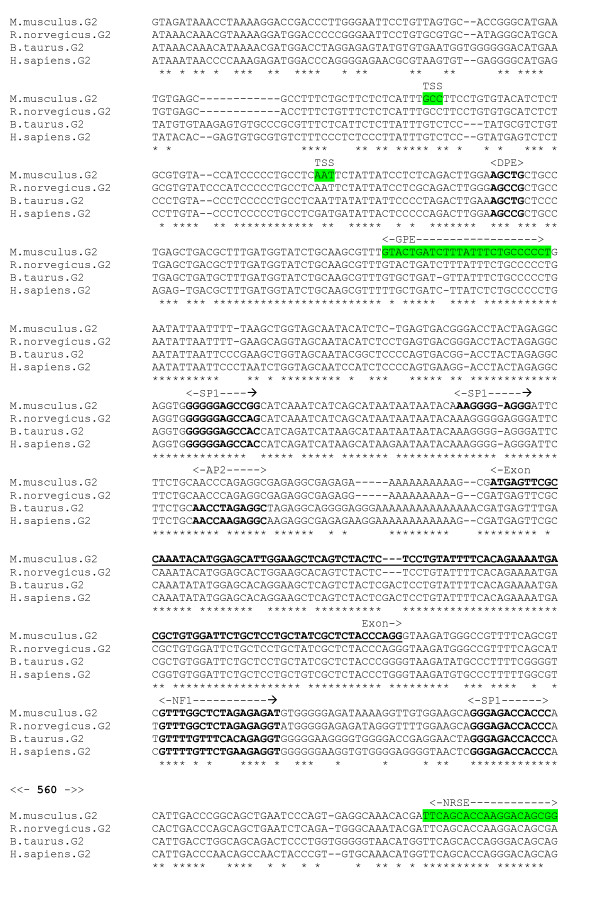
**γ2 gene promoter alignment**. Mouse exon underlined; TSS, GPE and NRSE (green). Putative promoter elements in bold.

An alignment of the promoter region for four species – human, cow, mouse and rat – reveals that the identified region maintains a very high level of conservation across all species downstream of the TSS (figure 12). Sequence identity is 88% in this region, but is not so high at the 5-end of the first major TSS. A number of putative TFBS sites, including NF1, AP2, and several SP1 sequences, are in conserved in the alignment downstream of the TSS sites. The experimentally determined NRSF and GPE elements [5] are also conserved in all species. The promoter appears to be without TATA box or INR; there exists however a conserved DPE sequence *AGC [TC]G *located at +27 bp with respect to the second the mouse major TSS.

### GABA_A _γ3 subunit gene

The γ3 subunit gene is expressed at low level in the cerebellum and hippocampus [33]. The gene promoter has not yet been experimentally determined. Analysis of the 2000 bp region upstream of the GenBank computationally-derived TSS for the human sequence by the PromoterInspector, McPromoter, NNPP and Promoter 2 programs gives a consensus promoter prediction within a region approximately 550–100 bp upstream of the GenBank putative TSS and CDS. This region is GC rich and contains no TATA sequences. The program CPGPlot predicts a 554 bp CpG island which matches closely the putative promoter region. The γ3 subunit gene promoter may therefore lie within a CpG island, in common with a number of other GABA_A_receptor subunit genes such as α2, α4, α5 and δ. Although such 5-end CpG islands commonly extend downstream of the promoter into the transcription unit [34], this does not appear to be the case for any of the GABA_A _subunit genes.

**Figure 13:**
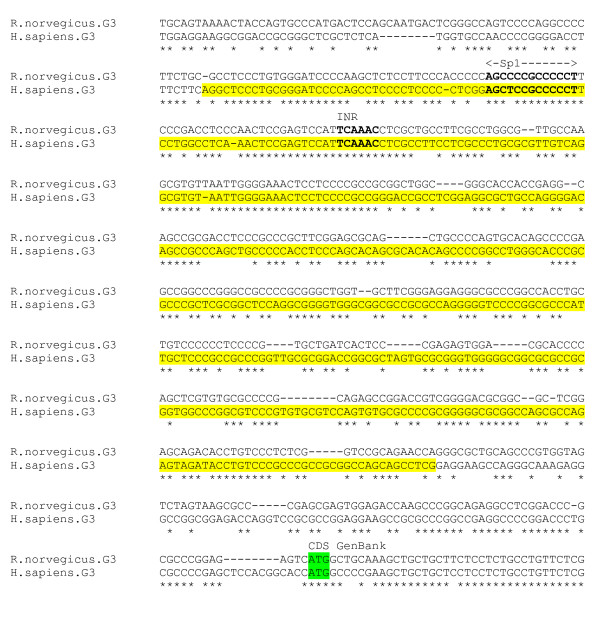
**γ3 gene promoter alignment**. Predicted human CpG island (yellow) and GenBank CDS (green). Putative promoter elements in bold.

An alignment of the region was generated using human and homologous mouse sequence (figure 13). The region contains numerous putative regulatory elements such as SP1, AP2 and NRSE, detectable by MatInspector etc; however these are generally not conserved in the alignment. An INR element *TCAAC *located 70 bp downstream of the start of the predicted CpG island is conserved.

### GABA_A _δ subunit gene

Expression of the GABA_A _receptor δ subunit gene is most abundant in granule cells of the cerebellum and dentate gyrus. The 5-end of the murine δ subunit gene contains a region with CpG island features, and lacks canonical promoter elements such as the TATA box [35]. The rat δ subunit gene and promoter has been characterised by Motejlek et al [3] who have mapped the major TSS, which is enclosed within an INR element *CCACTCT*. The promoter region lies within a CpG island. They have also identified a 22-bp purine-rich element, present in seven partially-overlapping copies, approximately 1800 nucleotides upstream of the TSS. The element is bound by a novel "brain-specific factor" (BSF1) that is present predominantly in cerebellar granule cells, correlating with GABA_A_δ subunit expression, however it is not clear if the factor plays any role in transcription regulation of the δ subunit gene.

**Figure 14:**
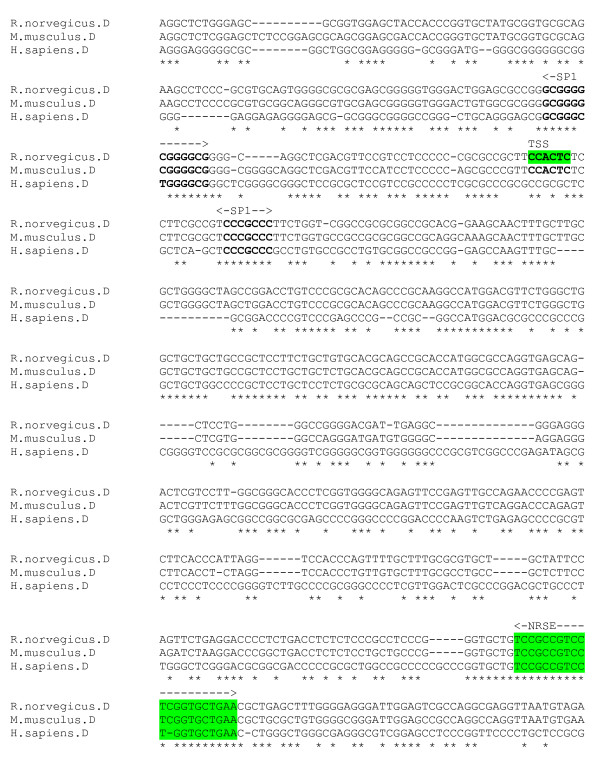
**δ gene promoter alignment**. Rat INR and downstream NRSE (green). Putative promoter elements in bold.

An alignment was produced using the genomic sequence encompassing the rat promoter region with upstream purine-rich BSF1 element repeats, and homologous human and mouse sequences. Whilst there is homologous sequence between species for the promoter region (figure 14), no sequence similar to the BSF1 element repeats is observable for the human and mouse. A functional negative strand NRSF binding site [5] exists downstream of the TSS in the first exon, *TCCGCCGTCCTCGGTGCTGAA*. This is conserved, with a single nucleotide deletion in the human sequence. The INR element enclosing the major TSS is not conserved in the human sequence. A number of putative SP1 sites are detectable in the region but generally these are not fully conserved in the alignment.

### GABA_A _ε subunit gene

Alternative splicing of the ε subunit gene yields a number of different transcript isoforms. Expression of the full protein sequence occurs abundantly only in discrete areas of the adult brain, such as the hypothalamus and locus ceruleus. In other tissues, truncated subunit protein sequences are transcribed [36]. The ε subunit gene promoter has not been characterised. The programs McPromoter, NNPP Cister and Promoter 2 predicted no promoter in the 2000 bp region upstream of the GenBank computationally-derived CDS for the human sequence GABA_A_ε subunit gene; however the program PromoterInspector predicted a 324 bp promoter within a region approximately 400 bp upstream of the GenBank computationally-derived CDS. For the mouse sequence, PromoterInspector also predicts a 200 bp promoter in a 180 bp upstream of the GenBank CDS.

**Figure 15:**
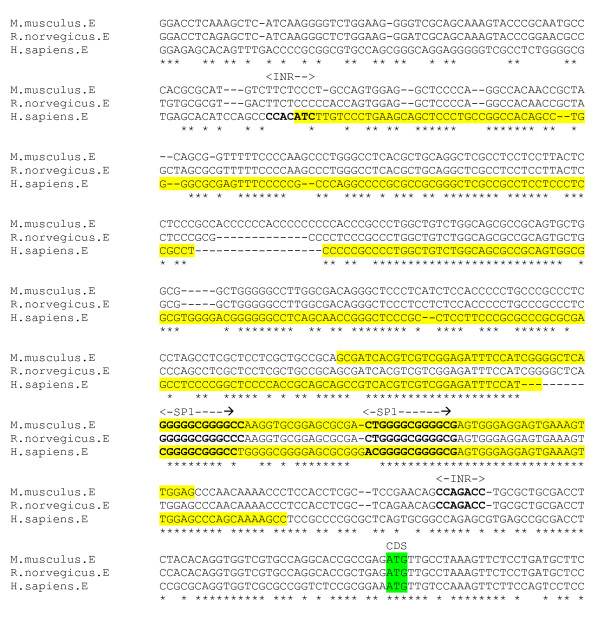
**ε gene promoter alignment**. Predicted human and mouse promoter regions (yellow) (MatInspector), and GenBank CDS (green). Putative promoter elements in bold.

The human predicted promoter region encompasses that of the mouse in a sequence alignment (figure 15). However, the use of cross-species sequence alignments to search for conserved transcription regulatory features is particularly problematic for the ε and θ subunits, because the genes from mouse and rat have been shown to display an unusually high level of divergence from their human homologues, and appear to be evolving at a faster rate than other GABA_A _receptor subunits [37]. The ε and θ subunit expression patterns and the functional properties of receptors containing these units may differ significantly between species. The transcription regulatory features will therefore probably be more variable between species than for the other subunits.

The putative region appears to contain no fully conserved canonical core promoter features, however the mouse and rat sequence have an INR motif *CCAGACC *45 bp upstream of the GenBank CDS, whereas the human sequence contains an INR motif at the beginning of the putative promoter region. For all species, the region has the features of a CpG island (CPGPlot), and contains conversed SP1 binding site sequences.

### GABA_A _θ subunit gene

GABA_A _receptor θ subunits show very similar distributions to ε subunits throughout the brain, and may also vary significantly between species [37]. Novel α3θε GABA_A _receptors with unique electrophysiological and pharmacological properties may form in monoaminergic neuronal cell-groups [38]. There is no experimental data for the θ subunit promoter. Promoter prediction programs were used to search both mouse and human sequences. No promoters were predicted by the MatInspector or McPromoter programs in either sequence. The program NNPP predicts two core promoters in the human sequence approximately 1500–1600 bp upstream of the GenBank TSS. Each putative promoter has a TATA motif. No promoter was predicted by NNPP in the homologous mouse sequence, and the TATA sequences are not conserved.

**Figure 16:**
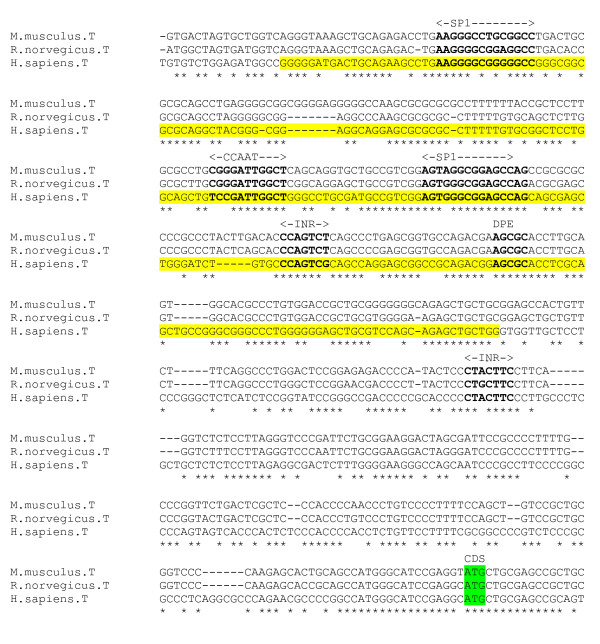
**θ gene putative promoter alignment**. Predicted human CpG islands (yellow, CpGPlot) and GenBank CDS (green). Putative promoter elements in bold.

Further downstream, CpG islands are predicted for both human and mouse (figure 16), 100–200 bp upstream of the GenBank TSS. This region contains a number of putative TFBS elements such as an negative CCAAT box and SP1 consensus sequences which are conserved in the alignment. There are also conserved consensus sequences for two INR elements, one of which has a downstream DPE sequence. Genes for ε and θ subunits from rodent species display a high level of divergence from their human homologues ([37]. Despite the expectation that the transcription regulatory features may therefore be more variable between species than for the other subunit genes, a high level of sequence identity was observed in the aligned putative promoter regions for these genes, with conservation of putative core promoter elements.

### GABA_A _π subunit gene

The subunit encoded by this gene is expressed in several non-neuronal tissues, and most abundantly in the uterus [39].

**Figure 17:**
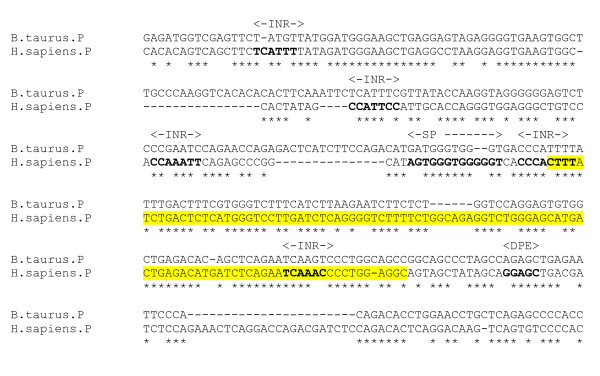
**π gene promoter alignment**. Predicted human promoter region (yellow, Promoter 2). Putative promoter elements in bold.

The π subunit gene promoter has not yet been experimentally determined. The programs PromoterInspector, McPromoter and NNPP predicted no promoters in the 2000 bp region upstream of the GenBank start point for the human GABA_A_π subunit gene sequence. Program Promoter 2 makes a marginal prediction (score 0.613) of a promoter between 200–300 bp upstream. There are several INR motifs in the human sequence 450 bp upstream of the gene start point, one of which with a DPE element at +28 from the putative TSS. However when an alignment was made with homologous sequence from the cow gene these motifs were not conserved (figure 17). Putative AP1 and SP1 sites found by the Cister program in the vicinity of this region of sequence were not conserved.

The 5-end of this gene displays some characteristics of a multiple start site promoter region such as that observed in the α2 subunit gene. Here also, there is no TATA sequence or well-defined TSS, but a number of INR motifs form a transcription initiation window of weak transcription start sites, which again are not fully conserved across species. Sequence identity in this region is lower than in the promoters of GABA_A _receptor genes with a single TSS, and individual start sites are not as well conserved. This appears to be characteristic of MSS promoters, and is presumably a result of relaxed selected pressure due to a degree of redundancy introduced by MSS transcription. It is quite possible also that the computationally derived GenBank gene origin is incorrect, and the TSS and promoter may lie outside this region of sequence.

### Transcriptional regulation of the GABA_A _receptor subunit gene cluster on chromosome 5

The human α1, β2, γ2 and α6 subunit gene cluster is located on chromosome 5q33 and spans 500–700 kb. The genes lie in the order β *2 *- α *6 *- α *1 *- γ *2*, with β2 and α6 in a head-to-head orientation. α6 high-level expression is restricted to cerebellar granule cells, and is probably regulated independently from the other genes in the cluster [18]. Wang et al [4] have determined that NFI-A factor is abundant in cerebellar granule cells and binds the α6 subunit promoter, playing a critical role in α6 gene expression. The γ2 subunit gene's expression profile overlaps with that of α1 and β2, but is yet more widespread, combining with several subtype variants in addition to the most common 2α *1 *- 2β *2 *- 1γ *2 *configuration. Transcriptional control of γ2 is therefore at least partially independent from that of α1 and β2. Its promoter is known to contain an NRSF binding site and a "Gamma Promoter Element", both of which direct expression in neuronal cells [5].

The α1 and β2 genes are widely expressed in neural tissue, and have almost identical expression profiles. The question therefore arises as to whether they have the same expression profiles by virtue of each possessing common transcriptional regulatory features in individual promoters, or whether coordinate regulation is achieved by the action of shared long-range transcriptional regulatory elements such as enhancers, silencers or an LCR. The α1 subunit gene core promoter is well characterised, and contains a canonical promoter [23], with TATA box, INR and DPE motifs, and an upstream SP1 site, typical of ubiquitously expressed housekeeper genes. There are also putative sites for NFI and NRSE in the α1 subunit gene, but there is no experimental evidence that they are required for tissue-specific expression (indeed, the NRSE motif is not conserved in cross-species alignments). Although the β2 subunit gene core promoter is undetermined, computational analysis here provides evidence that this subunit is under the control of a very similar promoter, with TATA box, INR, DPE core elements, also with an upstream SP1 site. Based upon this predicted promoter, the β2 subunit expression profile may closely matches that of the α1 subunit at least in part by possessing the same core promoter characteristics.

### Prediction of long-range regulatory features using comparative analysis

Both proximal and long-range regulatory features are more highly conserved between species by evolutionary selective pressure than is the surrounding non-regulatory DNA. Functional long-range regulatory features in a gene cluster should therefore stand out as relatively short regions of intergenic sequence which are more highly conserved than the surrounding background DNA. In order to investigate whether the α1 and β2 subunit genes share common long-range regulatory elements, the rVista program ([40] was used to perform cross-species, comparative analysis of the human chromosome 5 GABA_A _cluster locus, and intergenic homologous regions were isolated for further analysis.

**Figure 18:**
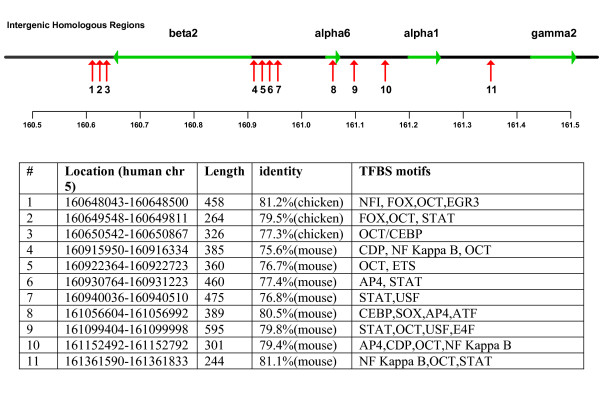
**Intergenic conserved sequences in Chr5 GABA cluster**. (a) Approximate location of conserved region. (b) Table showing Putative TFBSs. (Data from rVista).

Firstly the human and chicken DNA sequences were compared. A cluster of three conserved regions with sequence identity greater than 70% were identified between 3–5 kb from the end of the β2 gene (figure 18). The first of these regions (numbered 1 in figure 18), 458 bps with 81.2% identity, is particularly rich in putative sites for ubiquitous or neural tissue-associated TFBSs. It includes motifs for NFI, a regulator of the α6 subunit [4], OCT1, FOX (fork-head box) and EGR3, which is linked with α4 subunit promoter activity [26]. Given the location of the three human-chicken homologous intergenic sequences, 3–5 kb beyond the final β2 subunit gene exon, it is also possible that the high sequence identity indicates the presence of a degenerate or un-annotated alternative exon. For the third sequence, for example, a possible ORF extends from base 73 to 234 on the reverse (β2 gene) strand. However, as the region is rich in regulatory motifs it probably merits further analysis as a possible locus for regulation of one or more genes in the cluster. Several other regions of unknown function in the cluster were locally conserved between human and chicken, but were not enriched with TFBS motifs.

The rVista program was also used to compare human and mouse DNA in the GABA_A _cluster, to identify potential regulatory features limited to mammalian species. This revealed a number of intergenic homologous regions with sequence identity greater than 75%, not conserved in the chicken species. These were further analysed for putative neuronal or ubiquitous TFBS motifs. In addition to the motifs found in the conserved chicken homolog, these regions contain motifs for STAT (signal transducer and activator of transcription), CEBP (CCAAT/enhancer binding protein), CDP (CCAAT displacement protein), NF (Nuclear Factor) Kappa B, activator protein 4 (AP4), upstream stimulating factor (USF), activating transcription factor (ATF), enhancer factor 4 (E4F). An analysis of conserved intergenic regions is summarised in figure 18. Each of these conserved regions represent potential distal regulatory elements for one or more of the genes in the cluster, which could be inactivated by the neomycin gene insert [18] which was observed to cause disruption of α1 and β2 expression.

### Potential DNase I hypersensitive sites in the gene cluster

Regions of DNase I hypersensitivity within a gene locus, which are not associated with core promoters, are indicators of distal cis-regulatory elements such as enhancers or silencers [41]. Clusters of HSs provide markers for possible LCRs controlling transcription in the locus. As there is currently no HS prediction tool available, the locus of the GABA_A _chromosome 5 cluster was analysed based upon published locations of predicted HSs [41] (Coordinates are from the April 2003 human genome assembly). Although this data is based upon analysis of erythroid sequences, it was hypothesised that a proportion of the predicted sites would also be DNase I hypersensitive in other cell lines [41,42], including neuronal cells.

**Figure 19:**
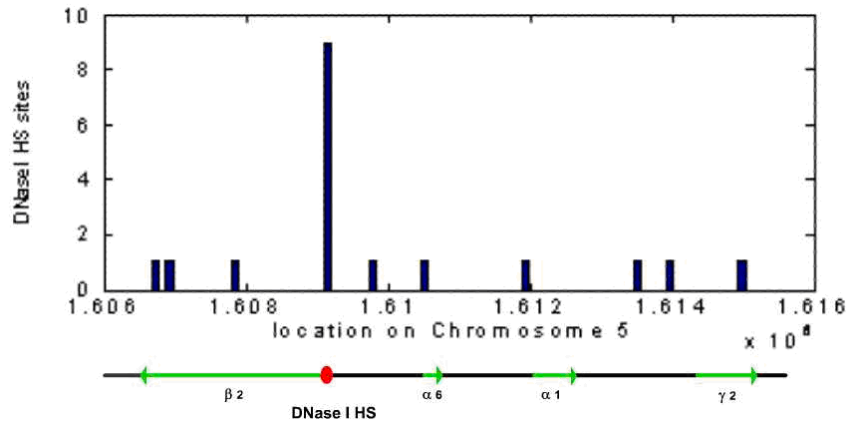
**Putative DNase I hypersensitive sites**. Distribution of predicted HSs in Chromosome 5 GABA receptor gene cluster (data from Noble et al [41]), and their locations in gene cluster.

An analysis of the gene cluster reveals that there are 18 predicted HSs within the 862 kb locus. The positions of the β2, α6 and α1 gene promoters in the cluster closely coincide with HS predictions, illustrating the potential value of this approach. Nine of the 18 predicted HSs conspicuously fall into a 2.5 kp region, which is contiguous apart from three 125 bp segments. This putative HS cluster, approximately 1.5 kb downstream from the β2 gene (on the anti-sense strand in figure 19), is a candidate site for a transcriptional regulatory region controlling one or more genes in the cluster. The proximity to the β2 promoter suggests a regulatory feature specific to this gene. Or, based upon the beta globin and other LCR models, these could be constitutive HSs clustered with other neuronal-specific HSs forming an LCR, augmenting transcription of both β2 and α1. HSs, like other transcriptional control sequences, tend to be conserved across species [42], and the organisation of the β-globin LCR, for example, is highly conserved in the human and the mouse gene locus [11]. There is no cross-species data available for HSs in the GABA_A _receptor gene cluster locus with which to perform comparative analysis. However, it is of interest to note that the predicted cluster of HSs corresponds closely with a cluster of four intergenic homologous regions identified by comparative analysis of human and mouse DNA (figure 18, numbered 4–7).

### Prediction of S/MARS and a model for coordinate regulation of β2 and α1 subunit genes

The program MAR-Wiz was used to predict S/MARS within and around the human chromosome 5 GABA_A _receptor gene cluster, and in the homologous mouse cluster on chromosome 11. Results with a cut-off score of 0.7 were considered.

**Figure 20:**
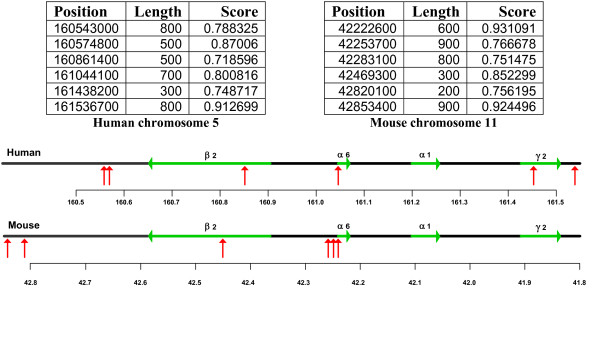
S/MAR predictions for human and mouse GABAA receptor gene cluster.

For both species, S/MARs are predicted in the vicinity of the α6 gene promoter region, and flanking the gene cluster (figure 20). This configuration, with a centrally located S/MAR and two S/MARs flanking the cluster, suggests the model similar to that proposed by Heng et al [17], in which S/MARs are selectively utilised to provide two functions: as structural anchor points for chromosomal attachment to the nuclear matrix, and as dynamic features which draw potentiated chromatin loops into transcriptional machinery in the proximity of the nuclear matrix. The model proposed here (figure 21) is also based partially upon that proposed for the multigenic human protamine chromatin domain, where in absence of a well-defined LCR, S/MARS bounding the domain act synergistically to regulate the expression of the gene cluster [15]:

(a) Firstly, the region surrounding the gene cluster is anchored to the nuclear matrix by flanking S/MARS, which establish a tissue-specific chromatin domain and define the boundaries of transcriptional activity by preventing the propagation of chromatin condensation, thus shielding the locus from the silencing effects of neighbouring chromatin. Tethering of the domain to the nuclear matrix may also bring it into proximity with chromatin modifying processes which participate in opening of the chromatin loop domain to potentiate transcription.

(b) A central, functional S/MAR in the vicinity of the α6 subunit gene promoter migrates to the nuclear matrix, under the control of accessory transcriptional regulator factors. This causes the potentiated chromatin to associate with transcriptional machinery proximal to the nuclear matrix. The location is approximately 100 kb from a region containing a cluster of predicted for HSs, which was also observed to be rich in conserved putative TFBSs upon comparative analysis of the human and mouse genomes. The central S/MAR may also play a role in the demethylation of DNA regions to trigger further chromatin conformational changes.

(c) The transcriptional machinery reels in the open chromatin in either direction until a promoter is encountered, at which point gene transcription initiates. Whilst the α6 and γ2 genes in the cluster are regulated independently by other cell-specific TFs, a shared complement of TFs and corresponding cis-regulatory elements presumably accounts for the identical expression profile of the β2 and α1 subunit genes. Distal enhancer elements, some of which are shared by both β2 and α1 subunit genes, may loop back into the region of the transcriptional machinery to provide tissue-specific transcriptional augmentation.

(d) Transcriptional elongation causes the functional S/MAR to dissociate from the vicinity of the nuclear matrix, so that once the transcription is complete, a new cycle of transcription can be initiated. Selection of either the β2 or α1 gene for transcription may be random, or there may be an unknown mechanism of alternating the expression of β2 and α1 to ensure parallel expression levels.

The model provides an explanation for number of observations – the head-to-head configuration of the β2 and α1 subunit genes, their parallel expression profiles, and the parallel down-regulation of expression observed upon insertion of the neomycin gene insert at exon 8 of the α6 subunit gene [18], which is the vicinity of the proposed functional S/MAR, and is presumably close enough to interact and to reduce its effectiveness. The basic model could also incorporate expression of the α6 subunit, assuming that additional gene-specific factors contribute to produce a different tissue-specific expression profile. However it is probable that the functional S/MAR would not be critical for γ *2 *gene transcription, since its expression is unaffected by the neomycin gene insert and subsequent S/MAR disruption.

## Conclusion

*In silico *comparative analysis of GABA_A _receptor subunit genes was performed to predict potential regulatory features and in particular to identify the means of coordinate regulation in the gene cluster comprising the α1, β2, γ2 and α6 subunits. Bioinformatics resources were used to generate a number of predictions which were substantiated by cross-species comparative analysis and are subject to wet-laboratory verification. Putative promoters were identified for the β2, γ1, γ3, ε, θ and π subunits, which may be experimentally verified by cloning of the identified DNA segment and screening for promoter activity using luciferase reporter gene assays. Putative core elements and proximal TFs were identified within these predicted promoters, and within the experimentally determined promoters of the other subunits.

A region of predicted DNase I hypersensitive sites within the GABA_A _receptor cluster on human chromosome 5 represents a candidate site for transcriptional regulatory features controlling one or more genes in the cluster. The experimental procedures RNA TRAP and 3C could be used to verify whether this region comes into contact with the DNA of the expressed genes whilst transcriptionally active, as described by established models for enhancer action [13].

Given the disparate orientations of the genes in this cluster, it seems unlikely that they are under the control of a canonical, orientation-dependent LCR. Based upon a putative promoter identified for the β2 subunit gene, it is possible that its expression profile closely matches that of the α1 subunit at least in part by possessing the same core promoter characteristics. The model proposed here for their coordinate regulation is based upon the selective use of S/MARS, in which the chromosomal sequence surrounding the gene cluster is first anchored to the nuclear matrix by flanking S/MARS to establish the boundaries of transcriptional activity, and further directed by another functional S/MAR. Spatial and temporal fine-control of transcriptional activity may be achieved by gene-specific factors and cis-regulatory elements, but other distal, intergenic putative regulatory elements were isolated and may be common to both α1 and β2 genes. The model accounts for a number of features of the gene cluster and its regulation, including the orientation of the genes, and disruption of α1 and β2 subunit gene transcription by the insertion of a neomycin gene in the close vicinity of the α6 gene, which is proximal to a critical S/MAR. A first step in verification of this model would be the use of Fluorescence in situ hybridisation (FISH) techniques to visualise the localisation of labelled predicted S/MARs on the nuclear matrix.

## Methods

### Core promoter prediction

For each GABA_A _receptor subunit, DNA sequence data was obtained from the NCBI website for analysis. In most cases, the sequences were the latest versions of genomic DNA from the Entrez Gene database. Where the promoter region or gene transcription start site is undetermined, the sequences are normally annotated in GenBank with an mRNA start point, derived by automated computational analysis, sometimes with supporting experimental evidence. The first 1000 base pairs upstream of the given 5'-end were typically used for initial analysis. This region of sequence was presumed to contain the core and promoter region and proximal regulatory elements. A number of promoter prediction programs were used, including PromoterInspector [43], NNPP [44], McPromoter [45], CISTER [46] and Promoter 2 [47]. These are popular, freely available program which each use different promoter-predicting methodologies – neural nets, HMMs and context-based approaches. Typically, several of these programs were used on the sequence to build up a consensus prediction for the core promoter region, which was then further analysed for individual regulatory features. Default options and parameters were used with these programs unless stated otherwise. To search for TFBSs in promoter regions, the Transcription Element Search System (TESS, [48]), P-MATCH ([49] and program MatInspector [9] were used. These programs each provide searches restricted by organism and tissue type; the search results were restricted to binding sites for vertebrate factors which are either ubiquitous or neuron-specific.

### Comparative promoter analysis

Comparative analysis can provide powerful evidence for the *in vivo *functionality of TF binding, and provides a means to eliminate false positives from the large result sets typically created by PWM-based TFBS prediction programs. Functional TF binding sites are likely to be situated in conserved non-coding regions, and furthermore, to be located in equivalent positions across genomes [12]. For each GABA_A _receptor subunit, sequences from several species were used to generate multiple sequence alignments of the gene promoter regions to identify conserved features. The alignments were performed using the CLUSTALW (1.81) Multiple Sequence Alignments program using default options unless stated otherwise.

The success of the comparative sequence analysis approach is largely dependent upon the selection of species which are at a suitable evolutionary distance. The sequence difference between closely related species will not provide any meaningful filtering of results, whilst comparison of highly unrelated species will be unlikely to reveal any conserved binding sites. In each case, sequences from human and mouse gene promoter regions were used with those for other selected available species. Sequence data for human and mouse is available for all GABA receptor subtypes, and the species are generally at a suitable evolutionary distance for the effective filtering of results. Sequence conservation between these species in non-coding regions was taken as additional evidence for the biological significance of predicted regulatory features. Quoted sequence identity scores were derived by using the *alistat *program provided as part of the HMMER 2.2 g HMM analysis package (hmmer.wustl.edu), using the Clustal X alignments as input. In most cases, only putative TF binding sites and promoter features which are largely conserved in all species in the alignment are reported in the results section. The filtering of TFBS predictions by species, cell specificity and finally by conservation in alignments reduces the number of predicted TFBSs to realistic levels, albeit at the risk of eliminating true positives from the result sets.

### Evidence for gene cluster co-regulatory features

Co-regulated genes may each possess the same promoter features, or they may share common regulatory features. Whilst analysis of the individual gene promoters can identify the former, prediction of long-range features responsible for the coordinate expression of several genes requires analysis of the whole gene cluster sequence. Regions of intergenic sequence which are more highly conserved than the surrounding background DNA would be strong candidates for such functional regulatory features. The rVista 2.0 program [40] was used to perform cross-species, comparative analysis of the human chromosome 5 GABA_A _cluster locus in order to identify potential intergenic regulatory elements in addition to the core promoters of individual genes. The program offers a combination of signal-based TFBS searches with comparative sequence analysis to reduce the number of false positive matches and to provide supporting evidence for site functionality.

### Predicting distal regulatory features as regions of DNase hypersensitivity

HSs occur over shorter stretches of DNA (typically ~250 bp), and are perhaps two orders of magnitude more sensitive to DNAse I than bulk chromatin [10]. Formation of hypersensitivity is a result of interaction of multiple transacting factors bound to cis-regulatory elements, and is taken as a reliable indicator of functional transcriptional features, such as promoters and enhancers, in non-coding DNA [41].

Noble et al [41] used a Support Vector Machine (SVM) to recognize HSs in genomic sequence data. The SVM was used to predict HSs for all non-repetitive sequence in the human genome, partitioned into 225 bp segments. High-scoring positive-predicted values in subsequent experimental validation of the predicted HSs suggested to the authors that "elements identified by the SVM might represent a class of HSs that are active in many tissues or are even constitutive." [41]. Crawford et al [42] have used high-throughput experimental analysis to identify clusters of HSs in CD4+ T cells (lymphocytes) for a representative sample of sequences from the human genome. Whilst 10% of the HSs thus identified are only detectable in lymphocytes, the remaining 90% were confirmed as HSs for all tested cell types. Whilst there is as yet no generally available tool for predicting HSs, this data suggests that a high percentage of sites of DNase I hypersensitivity in one tissue type could also be so in other tissue types, including neuronal tissue. Based upon this hypothesis, the predicted HSs [41] were analysed as possible markers for LCRs and other distal regulatory elements such as enhancers or silencers, controlling transcription in the locus of the chromosome 5 GABA_A _gene cluster.

### Prediction of scaffold/matrix associated regions

S/MARS are found either in non-transcribed regions, at the borders of chromatin domains, or in close association with non-coding transcription elements such as enhancers or introns. S/MARs themselves are often rich in TF binding sites, with a local over-representation of specific AT-rich motifs. [50,51]. MAR-Wiz  is a web-based tool for predicting S/MARs based upon a number of analysis rules including origin of replication, TG-richness, curved DNA, kinked DNA, and topoisomerase II recognition. MAR-Wiz was used to predict S/MAR regions in the gene cluster locus, as potential anchor points delimiting chromatin loop domains.

The computational resources which were used in this paper are summarised in table 1.

**Table 1:** Gene Regulation Prediction Software utilised

**Resource**	**Method/Description**	**Web Address**
CPGPLOT	CpG island detection	
TESS, Transcription Element Search System	PSSM-based TF binding site search	
Matinspector	PSSM-based TF binding site search	
MATCH and P-MATCH	PSSM-based TF binding site search	
TSSG	TSS Prediction	
NNPP, Neural Network Promoter Prediction	Uses Neural Networks to predict basal promoter region and TSS.	
Promoter Scan	TSS prediction, limited to TATA class promoters	
Promoter 2.0	Uses Neural Networks with Genetic Algorithms to predict vertebrate PolII promoters regions.	
PromoterInspector	Context-based prediction of eukaryotic pol II promoter regions.	
McPromoter	Markov Chain/Neural net based Promoter and TSS prediction program	
MAPPER	HMM based cis-element search, lists interacting TFs (promoter modules) and TF class summary	
NCBI Entrez Gene	Searchable database of genes	
Cister, Cis-element Cluster Finder	HMM based cis-element cluster search	
RVista 2.0	Phylogenetic footprinting, combines database searches with comparative sequence analysis.	
MAR-Wiz	S/MAR motif sequence search	

## Abbreviations

3C Chromosome Conformation Capture

AP4 Activator protein 4

ATF Activating transcription factor

BSF1 Brain specific factor 1

CDP CCAAT displacement protein

CDS Coding Sequence

CEBP CCAAT/enhancer binding protein

CNS Central nervous system

CREB cAMP-responsive element binding protein

DPE Downstream promoter element

E2F, E4F enhancer factor 2, 4

EGR3 Early growth response factor 3

ERE Estrogen response element

FISH Fluorescence in situ hybridization

FOX Fork-head box

GABA Gamma-aminobutyrate

GABA_A _GABA receptor type A

GRE Glucocorticoid response element

HMM Hidden Markov model

HS DNAse I hypersensitive site

IK2 Ikaros factor

INR Initiator

LCR Locus Control Region

LGIC Ligand-gated ion channel

MSS Multiple start site

nAChR Nicotinic acetylcholine receptor

NFI, NF-1 Nuclear factor I family of transcription factors (CAAT box binding site)

NRSE, NRSF Neuron-restrictive silencer element/factor

ORF Open reading frame

OCT1 Octamer binding protein

PIC Pre-Initiation complex

PGK-Neo Phosphoglycerine kinase neomycin resistance hybrid gene

PWM Positional weight matrix

REST RE1 silencing transcription factor

S/MAR Scaffold/Matrix Attachment Region

SP1,2 etc Specifity protein 1,2

STAT Signal transducer and activator of transcription

SVM Support vector machine

TF Transcription factor

TFBS Transcription factor binding site

TRAP Tagging and recovery of associated proteins

TSS Transcription start site

USF Upstream stimulating factor

## Authors' contributions

CJJ carried out background research, genomic analysis and preparation of manuscript.
